# Validation study of candidate single nucleotide polymorphisms associated with left ventricular hypertrophy in the Korean population

**DOI:** 10.1186/s12881-015-0158-1

**Published:** 2015-03-15

**Authors:** Jin-Kyu Park, Mi Kyung Kim, Bo Youl Choi, Yusun Jung, Kyuyoung Song, Yu Mi Kim, Jinho Shin

**Affiliations:** Division of Cardiology, Department of Internal Medicine, Hanyang University College of Medicine, Seoul, South Korea; Department of Preventive Medicine, Hanyang University College of Medicine, Seoul, South Korea; Department of Biochemistry and Molecular Biology, University of Ulsan College of Medicine, Seoul, Korea; Department of Preventive Medicine, Dong-A University College of Medicine, Busan, South Korea

**Keywords:** HyperGEN study, Left ventricular hypertrophy, Single nucleotide polymorphism, Korean population

## Abstract

**Background:**

Left ventricular hypertrophy (LVH) is a valid predictor for cardiovascular mortality and morbidity regardless of age, gender, and race. The HyperGEN study conducted a genome-wide association study and identified twelve single nucleotide polymorphisms (SNPs) associated with LVH. The aim of this study was to validate these candidate SNPs in the Korean population.

**Methods:**

Among 1637 individuals from the Korean Multi-Rural Communities Cohort Study (MRCohort) of the Korean Genome Epidemiology Study (KoGES), we carried out a linear regression analysis with left ventricular mass index (LVMI) and a logistic regression analysis for LVH status.

**Results:**

The rs4129218 on chromosome 12 tended to be associated with LVM/body surface area (adjusted β = −0.023; p = 0.036) and LVM/height^2.7^ (adjusted β = −0.027; p = 0.016), and was marginally protective against LVH after adjustment for age, sex, body mass index, serum creatinine, systolic blood pressure, heart rate and antihypertensive medication (adjusted odds ratio = 0.766 and 0.731; p = 0.027 and 0.007 according to indexation by BSA and height^2.7^, respectively).

**Conclusions:**

In the Korean population, the minor allele of rs4129218 had borderline association with lower LVM. This study suggests that rs4129218 on chromosome 12 showed consistent tendency of possibly related loci for LVH independent of ethnic background.

**Electronic supplementary material:**

The online version of this article (doi:10.1186/s12881-015-0158-1) contains supplementary material, which is available to authorized users.

## Background

Left ventricular hypertrophy (LVH) is a well-known risk factor for cardiovascular morbidity and mortality. Increased LV wall thickness predicts cardiovascular disease events [1], and dilatation of LV predicts congestive heart failure [2]. LVH and increased LV mass (LVM) are risk factors of coronary artery disease, congestive heart disease and stroke [3-7].

LVM is influenced by multifactorial traits, such as blood pressure (BP), obesity, body size, and gender [8-12], which can explain only one-half to two-thirds of the inter-individual variability of LVM. Additionally, genetic susceptibility is expected to be associated with degree of hypertrophy regardless of race [13-17]. Several lines of evidence have indicated that echocardiographic LV geometry and LVH show heritability [13,14,18-20]. Recently, genome-wide association studies (GWAS), such as the HyperGEN study have identified several SNPs associated with echocardiography LVH in Western populations [21].

However, the single nucleotide polymorphisms (SNPs) suggested in the HyperGEN study have not been validated for Asian populations with different lifestyles, environmental backgrounds and genetic differences [16]. Although a previous study performed a GWAS for electrocardiographic LVH in Asian populations [22], no report concerning the genetic association of echocardiographic LVH has been published. Therefore, we conducted a validation study of candidate SNPs reported in the HyperGEN study in relation to LVH in the Korean population.

## Methods

### Study subjects

The Yang-pyeong cohort is part of the Korean Multi-Rural Communities Cohort Study (MRCohort) of the Korean Genome Epidemiology Study (KoGES), which began in 2005. A total of 1841 men and women aged ≥44 years living in Yang-pyeong County in Korea were voluntarily recruited. Among them, we included 1745 subjects whose echocardiographic data were available in the analysis and excluded 108 subjects who had poor acoustic image quality, an interrogation angle >10°, any regional wall motion abnormalities, grade II or greater valvular regurgitation, any valvular stenosis, pericardial disease, cardiomyopathy on echocardiography, and atrial fibrillation. Finally, 1637 subjects (men 671 and women 966) were included in the study. The study was conducted according to the guidelines of the Declaration of Helsinki, and all procedures involving human subjects were approved by the institutional review board of Hanyang University. Written informed consent was obtained from all subjects.

### Echocardiographic measurement

All tests were performed by a single sonographer using a commercially available machine, (HP SONOS 2500; Hewlett-Packard, Inc., Andover, MA, USA) using a 2.5/2.0-MHz transducer. The sonographer was blinded to the patient’s clinical data. Images were obtained in the supine and left lateral positions, in the parasternal long-axis and short-axis views, and in the apical four- and two-chamber views. Left ventricular measurements were performed at or just below the mitral valve tips, by the leading edge-to-leading edge method, according to the recommendations of the American Society of Echocardiography (ASE) [23], with the M-mode guided by the two-dimensional examination. LV systolic and diastolic diameters, as well as interventricular septal and posterior wall thickness were measured at end-diastole, which was defined by the beginning of the QRS complex. LVM was calculated using the corrected ASE formula proposed by Devereux et al.

LVM = 1.04 × [(LVID + PWT + IVST)^3^ ‐ LVID^3^] × 0.8 + 0.6 [24].

Where, LVID, PWT, and IVST represent the LV internal dimension (cm), LV posterior wall thickness (cm) and interventricular septal thickness (cm), respectively. LVM was indexed for body surface area (LVM/BSA, g/m^2^), which was calculated by the Dubois formula, and for height^2.7^ (LVM/height^2.7^, g/m^2.7^), as recommended by De Simone et al. We set cut-off values for LVH as LVM/BSA ≥ 116 g/m^2^ in males and ≥ 96 in females and LVM/height^2.7^ ≥ 49 g/m^2.7^ in males and ≥ 45 g/m^2.7^ in females [25].

### General characteristics, anthropometrics, and biochemical variables

Subjects were interviewed by trained interviewers using a structured questionnaire to determine general characteristics, including age and antihypertensive medication. Height was measured using a standard height scale to the nearest 0.1 cm, and weight was measured with a metric weight scale to the nearest 0.01 kg in light clothing without shoes. Body mass index (BMI) was calculated by dividing the weight (kg) by height (m^2^). Waist circumference (WC) was measured halfway between the lowest rib margin and the iliac crest. We measured seated BP from the right arm by auscultation using a standard sphygmomanometer and a standard cuff. Two consecutive measurements of BP were taken after each subject had been sitting for at least 5 min. Systolic and diastolic BP were recorded to the nearest 2 mmHg. If the two systolic or diastolic BP readings were more than 5 mmHg apart, an additional measurement was performed, and the mean value of the closest two measurements was used for the subsequent analyses. Blood samples were collected in the morning after at least 8 h of fasting. Plasma total cholesterol, triglyceride, glucose and high-density lipoprotein cholesterol levels were measured with an ADVIA1650 Automatic Chemistry Analyzer (Siemens, New York, NY, USA).

### SNP selection and genotyping

Twelve SNPs from the HyperGEN study were included. Before genotyping candidate SNPs, three SNPs from the HyperGEN study (rs1833534, rs4129000 and rs238688) were filtered out because they had a minor allele frequency (MAF) lower than 0.05 in the Chinese and Japanese HapMap database [26]. Finally, nine SNPs were included for genotyping in the present study. Genotyping was performed with the use of Sequenom iPLEX (San Diego, USA) at the Analytical Genetics Technology Centre, Princess Margaret Hospital/University Health Network in Toronto, Canada. Polymerase chain reaction (PCR) primers were designed in a region of approximately 100 base pairs around the SNP of interest and an extension primer was designed immediately adjacent to the SNP. After PCR amplification, Shrimp Alkaline Phosphatase (SAP) was added, along with the primer extension mixture after a brief incubation. After a standardized PCR program, SpectroCLEAN resin was added to the mixture to prepare it for spotting and detection of the PCR products using a 384-well SpectroChip® and a Compact TM MALDI-TOF mass-spectrometer automatically.

### Statistical analysis

The descriptive analysis was performed using SPSS (Statistical Package for the Social Sciences) software (version 19.0; SPSS Inc., Chicago, Illinois, USA). The examined phenotypes comprised the continuous variables of LVM/BSA and LVM/height^2.7^, and the categorical phenotype of LVH, which was defined as aforementioned cut-off value [25]. LVMI was transformed into a natural log value to normalize the trait distribution. Multiple linear and logistic regressions with a dominant model were performed using PLINK 1.07 software (http://pngu.mgh.harvard.edu/~purcell/plink) [27], adjusting for age, gender, BMI, serum creatinine, systolic BP, heart rate, and antihypertensive medication. The HWE tests were conducted using PLINK. A *P* value <0.0056 (0.05 with 9 SNPs) was deem statistically significant to account for multiple testing.

## Results

The general characteristics of study subjects are shown in Table 1. The mean age was 61.2 ± 10.4 years, and the proportion of men was 41.0%. The study subjects had a somewhat high BMI and WC (mean BMI, 24.7 ± 3.3 kg/m^2^; mean WC, 87.8 ± 8.3 cm). Systolic and diastolic BP were 124.3 ± 17.7 mmHg and 79.9 ± 10.6 mmHg, respectively. The prevalence of hypertension was 44.3% and 483 (29.5%) subjects had taken antihypertensive medication. Echocardiographic data are presented in Table 2. The mean LVM/BSA and LVM/height^2.7^ were 95.6 ± 22.5 g/m^2^ and 45.4 ± 11.9 g/m^2.7^, respectively. Four hundred ninety one (30.0%) and 644 (39.3%) subjects were diagnosed with LVH according to indexation by BSA and height^2.7^, respectively.

**Table 1:** **Demographic and clinical characteristics of the study population**

**Characteristics**	**Total**	**Men**	**Women**	**P value**
	**(n = 1637)**	**(n = 671)**	**(n = 966)**	
Age, years	61.2 ± 10.4	61.8 ± 10.2	60.7 ± 10.5	0.040
Body mass index, kg/m^2^	24.7 ± 3.3	24.2 ± 3.1	25.0 ± 3.4	<0.001
Waist circumference, cm	87.8 ± 8.3	88.3 ± 7.9	87.5 ± 8.5	0.078
Systolic blood pressure, mmHg	124.3 ± 17.7	125.8 ± 17.2	123.2 ± 18.0	0.004
Diastolic blood pressure, mmHg	79.9 ± 10.6	81.5 ± 11.0	78.8 ± 10.1	<0.001
Pulse pressure, mmHg	44.4 ± 13.1	44.4 ± 12.5	44.5 ± 13.4	0.855
Heart rate, bpm	69.7 ± 10.1	69.2 ± 10.3	70.0 ± 10.0	0.135
Fasting glucose, mmol/l	5.7 ± 1.4	5.8 ± 1.4	5.7 ± 1.4	0.050
Total cholesterol, mmol/l	5.14 ± 0.96	4.92 ± 0.91	5.29 ± 0.98	<0.001
Triglyceride, mmol/l	1.76 ± 1.10	1.89 ± 1.23	1.68 ± 0.98	<0.001
High-density lipoprotein cholesterol, mmol/l	1.19 ± 0.28	1.16 ± 0.30	1.21 ± 0.27	<0.001
Serum creatinine, μmol/l	85.7 ± 15.91	95.5 ± 13.3	78.7 ± 14.1	<0.001
Hypertension, n (%)	726 (44.3%)	286 (42.6%)	440 (45.5%)	0.245
Antihypertensive medication, n (%)	483 (29.5%)	157 (23.4%)	326 (33.7%)	<0.001

Data are presented as mean ± SD unless otherwise indicated.

**Table 2:** **Echocardiographic data**

	**Total**	**Men**	**Women**	**P value**
	**(n = 1637)**	**(n = 671)**	**(n = 966)**	
LVID, cm	4.89 ± 0.46	5.02 ± 0.44	4.79 ± 0.45	<0.001
LVWT, cm	1.80 ± 0.24	1.87 ± 0.22	1.75 ± 0.24	<0.001
LVM, g	155.1 ± 40.0	167.0 ± 38.6	144.7 ± 37.6	<0.001
LVM/BSA, g/m^2^	95.6 ± 22.5	98.3 ± 20.8	93.7 ± 23.4	<0.001
LVM/height^2.7^, g/m^2.7^	45.4 ± 11.9	43.9 ± 10.2	46.5 ± 12.9	<0.001
Prevalence of LVH, n (%)				
Indexation by BSA	491 (30.0%)	116 (17.3%)	375 (38.8%)	<0.001
Indexation by height^2.7^	644 (39.3%)	179 (26.7%)	465 (48.1%)	<0.001

Data are presented as mean ± SD unless otherwise indicated.

Abbreviations: LVID, left ventricular internal dimension; LVM, left ventricular mass; LVM/BSA, left ventricular mass indexed by body surface area; LVM/height^2.7^, left ventricular mass indexed by height^2^; LVH, left ventricular hypertrophy; LVWT, left ventricular wall thickness.

**Figure 1:**
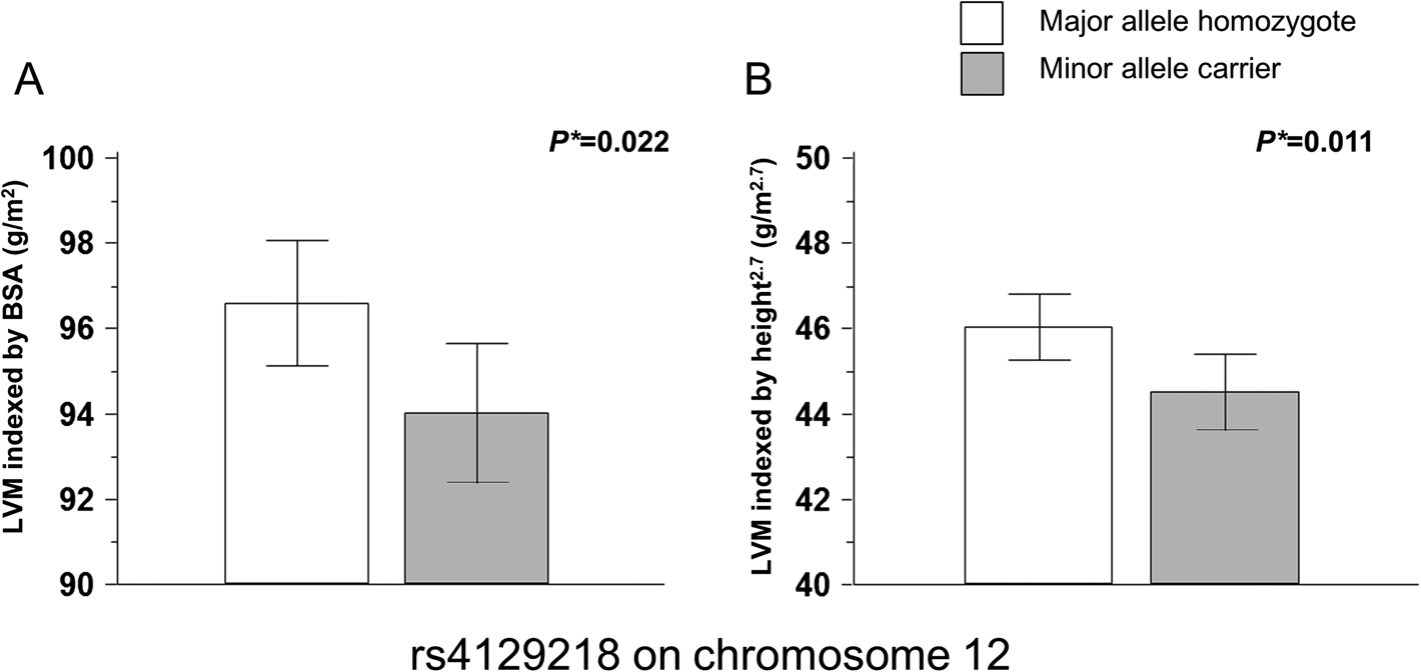
**Effect sizes of rs4129218 on two left ventricular mass index associations in Korean subjects.** (**A**, The mean value of LVM indexed by body surface area. **B**, The mean value of LVM indexed by height2.7). *Student’s t test. Error bars indicate 95% confidence intervals. White box, major allele homozygote; gray box, minor allele carrier; LVM, left ventricular mass.

Table 3 lists the nine candidate SNPs associated with echocardiographic LVH from the HyperGEN study. The SNPs examined did not significantly deviate from Hardy-Weinberg equilibrium. In Tables 4 and 5, one SNP (rs4129218 on chromosome 12) among the nine candidate SNPs from the HyperGEN study had a marginally negative linear correlation with LVM/BSA (adjusted beta = −0.023; 95% CI, −0.044–−0.001; *P* = 0.036) and LVM/height^2.7^ (adjusted beta = −0.027; 95% CI, −0.048–−0.005; *P* = 0.016) and its effect sizes are shown in Figure 1. This SNP, located in the *LOC100507065* gene (Table 3), tended to show a protective effect on LVH defined as LVM/BSA (adjusted OR = 0.766; 95% CI, 0.605–0.970; *P* = 0.027) and LVM/height^2.7^ (adjusted OR = 0.731; 95% CI, 0.582–0.919; *P* = 0.007). However, the *P* values did not meet statistical threshold (*P* <0.0056). In subgroup analysis, these findings were consistently observed in hypertensive subjects (Additional file 1: Table S1B and S2B, *P* = 0.035 and 0.021, respectively). Another SNP, rs6450415 on chromosome 5 also revealed an association with LVH defined as LVM/BSA (adjusted OR = 1.463; 95% CI, 1.148–1.865; *P* = 0.002) and LVM/height^2.7^ (adjusted OR = 1.320; 95% CI, 1.041–1.673; *P* = 0.022). However, no significant associations were noted in quantitative analysis (p = 0.107 and 0.178, respectively). In subgroup analysis, an association between rs6450415 and LVH was remained in only non-hypertensive subjects (Additional file 1: Table S1A and S2A, *P* = 0.002 and 0.013, respectively) but, not in hypertensive subjects (Additional file 1: Table S1B and S2B, *P* = 0.174 and 0.567, respectively).

**Table 3:** **Genotyping of nine SNPs from the HyperGEN study in the Korean population**

**SNP**	**Chromosome**	Position*	**Near Gene**	**Location**	**Minor allele**	**MAF**	**HWE**
rs409045	5	34,628,522	*RAI14*		C	0.129	0.499
rs6450415	5	57,053,058	*MIER3*		C	0.172	0.858
rs6961069	7	80,589,645	*CD36*		C	0.291	0.185
rs10499859	7	80,629,494	*CD36*	Intron	G	0.296	0.288
rs4129218	12	65,564,881	*LOC100507065*		G	0.227	0.353
rs1155635	13	70,373,320			G	0.423	0.958
rs2415872	14	44,638,258	*LOC101927351*	Intron	C	0.328	0.541
rs756529	20	49,394,471	*KCNB1*	Intron	G	0.391	0.56
rs10483186	22	34,943,863	*RP1-272 J12.1*	Intron	T	0.384	0.736

*dbSNP positions are given according to National Center for Biotechnology Information.

Map Viewer Build 37.

Abbreviations: HWE, Hardy-Weinberg equilibrium; LVMI, left ventricular mass index; MAF, minor allele frequency; SNP, single nucleotide polymorphism.

**Table 4:** **Association analyses between left ventricular mass indexed by BSA and nine candidate SNPs from the HyperGEN study in the Korean population**

		**Log (LVM/BSA)**				**LVH**			
**SNP**	**LVM/BSA, g/m** ^**2**^	**Beta§**	**L95**	**U95**	***P*** **value**	**OR§**	**L95**	**U95**	***P*** **value**
rs409045		−0.023	0.048	0.001	0.060	0.787	0.601	1.031	0.083
TT (n = 1239)	95.9 ± 22.5								
CT + CC (n = 398)	94.3. ± 22.3								
rs6450415		0.018	−0.004	0.041	0.107	1.463	1.148	1.865	0.002
TT (n = 1118)	95.2 ± 22.5								
CT + CC (n = 519)	96.4 ± 22.3								
rs6961069		0.001	−0.020	0.022	0.932	1.190	0.946	1.496	0.137
TT (n = 808)	95.5 ± 21.8								
CT + CC (n = 829)	95.6 ± 23.1								
rs10499859		0.001	−0.020	0.022	0.945	1.180	0.938	1.484	0.156
AA (n = 799)	95.6 ± 21.8								
AG + GG (n = 838)	95.4 ± 23.1								
rs4129218		−0.023	−0.044	−0.001	0.036	0.766	0.605	0.970	0.027
AA (n = 975)	96.6 ± 23.0								
AG + GG (n = 662)	94.0 ± 21.5								
rs1155635		−0.002	−0.025	0.020	0.831	0.905	0.710	1.153	0.420
AA (n = 543)	95.8 ± 22.5								
GA + GG (n = 1081)	95.5 ± 22.5								
rs2415872		−0.013	−0.034	0.008	0.236	0.855	0.680	1.075	0.181
GG (n = 735)	96.2 ± 22.4								
GC + CC (n = 902)	95.0 ± 22.5								
rs756529		0.015	−0.007	0.037	0.173	1.234	0.972	1.568	0.085
AA (n = 609)	94.4 ± 21.7								
GA + GG (n = 1028)	96.3 ± 22.9								
rs10483186		−0.006	−0.027	0.016	0.586	0.999	0.790	1.264	0.996
GG (n = 622)	96.2 ± 23.0								
GT + TT (n = 1015)	95.2 ± 22.1								

§Analysis adjusted for age, gender, body mass index, serum creatinine, systolic blood pressure, heart rate and antihypertensive medication.

Abbreviations: L95 and U95, confidence interval lower and upper 95%; LVH, left ventricular hypertrophy defined as LVM/BSA ≥ 116 g/m^2^ in males and ≥ 96 g/m^2^ in females; LVM/BSA, left ventricular mass indexed by body surface area; SNP, single nucleotide polymorphism.

**Table 5:** **Association analyses between left ventricular mass indexed by height** ^**2.7**^ **and nine candidate SNPs from the HyperGEN study in the Korean population**

		**Log (LVM/height** ^**2.7**^ **)**				**LVH**			
**SNP**	**LVM/height** ^**2.7**^ **, g/m** ^**2.7**^	**Beta§**	**L95**	**U95**	***P*** **value**	**OR§**	**L95**	**U95**	***P*** **value**
rs409045		−0.024	−0.049	0.001	0.062	0.816	0.629	1.058	0.125
TT (n = 1239)	45.5 ± 12.0								
CT + CC (n = 398)	45.2 ± 11.8								
rs6450415		0.016	−0.007	0.039	0.178	1.320	1.041	1.673	0.022
TT (n = 1118)	45.3 ± 11.9								
CT + CC (n = 519)	45.8 ± 11.9								
rs6961069		0.002	−0.019	0.024	0.840	1.157	0.927	1.445	0.197
TT (n = 808)	45.4 ± 11.3								
CT + CC (n = 829)	45.5 ± 12.5								
rs10499859		0.002	−0.019	0.024	0.838	1.176	0.942	1.469	0.152
AA (n = 799)	45.3 ± 11.3								
AG + GG (n = 838)	45.5 ± 12.5								
rs4129218		−0.027	−0.048	−0.005	0.016	0.731	0.582	0.919	0.007
AA (n = 975)	46.1 ± 12.2								
AG + GG (n = 662)	44.5 ± 11.5								
rs1155635		−0.004	−0.027	0.019	0.717	0.873	0.690	1.106	0.261
AA (n = 543)	45.4 ± 11.6								
GA + GG (n = 1081)	45.5 ± 12.1								
rs2415872		−0.011	−0.033	0.010	0.293	0.937	0.750	1.171	0.569
GG (n = 735)	45.8 ± 12.0								
GC + CC (n = 902)	45.1 ± 11.9								
rs756529		0.014	−0.008	0.036	0.218	1.059	0.842	1.332	0.625
AA (n = 609)	44.8 ± 11.5								
GA + GG (n = 1028)	45.8 ± 12.1								
rs10483186		−0.005	−0.027	0.017	0.637	0.943	0.752	1.184	0.614
GG (n = 622)	45.7 ± 12.0								
GT + TT (n = 1015)	45.3 ± 11.9								

§Analysis adjusted for age, gender, body mass index, serum creatinine, systolic blood pressure, heart rate and antihypertensive medication.

Abbreviations: L95 and U95, confidence interval lower and upper 95%; LVH, left ventricular hypertrophy defined as LVM/ height^2.7^ ≥ 49 g/m^2.7^ in males and ≥ 45 g/m^2.7^ in females; LVM/height^2.7^, left ventricular mass indexed by height^2.7^; SNP, single nucleotide polymorphism.

## Discussion

In this study, we successfully analyzed nine of the 12 candidate SNPs from the HyperGEN study in the Korean population. Among the nine candidate SNPs, two SNPs (rs6450415 on chromosome 5 and rs4129218 on chromosome 12) showed borderline association with LVH. Among them, only rs4129218 had marginal association with two types of LVMI in a quantitative trait study.

To our knowledge, the present study is the first replication study concerning SNPs for echocardiographic LVH in an Asian population. A previous study identified several candidate SNPs via GWAS for LVH in the Korean population [22], which was a study on electrocardiographic LVH. Although LVH using electrocardiography showed a stronger correlation with the genetic signal than LVH using echocardiography (39–41% versus 21–29%) [28], the electrocardiographic LVH and echocardiographic LVH predicted mortality independently and carried different prognostic information [29]. Therefore, SNPs associated with echocardiographic LVH may be different from SNPs associated with electrocardiographic LVH.

Arnett DK et al. demonstrated that three SNPs including rs409045 on chromosome 5, rs4129000 and rs4129218 on chromosome 12 showed a significant association with log-transformed LVMI in Caucasian subjects (*P* = 0.010, 0.035, and 0.007, respectively), and only rs4129218 tended to be significant for LVH (*P* = 0.038). In the present study, rs4129218 showed a marginal association with LVH, consistent with that in Caucasians. In the validation study for African American subjects, they reported associations of another two SNPs (rs238688 and rs756529 on chromosome 20) with log-transformed LVMI or LVH. In the present study, the association of rs756529 was not significant and rs238688 was monomorphic. There was no common finding between Caucasian and African American subjects in the HyperGEN study. GWAS was performed only in Caucasians, which likely led to different results in Caucasians and African Americans. Furthermore, this inconsistent finding may have resulted from different allele frequencies between the two ethnic subjects, and other risk factors for LVH may also have differed between subjects.

Rs4129218 is located on *LOC100507065,* uncharacterized lincRNA (large intergenic non-coding RNA), between *MSRB3* (methionine sulfoxide reductase B3) at ~98 kb and *RPSAP52* (ribosomal protein SA pseudogene 52) at 193 kb away. Although the two loci in intergenic or lincRNA regions do not exhibit biological functions, consistent associations in Caucasian data suggest important roles of these loci in LVID and LVH independent of ethnicity [30]. Recently, several studies demonstrated that vitamin D receptor (VDR) gene polymorphism is associated with LVM and predicts LVH progression in end-stage renal disease patients [31,32]. Notably, the VDR gene is located on chromosome 12. In view of these observations it could be worthy to consider if a linkage could exist between these genetic loci on chromosome 12. However, further investigation may be needed to verify this possibility.

Among other eight SNPs, the rs765529 on chromosome 20 was intragenic, which is located in the potassium voltage-gated channel, Shab-related subfamily, member 1 gene (*KCNB1*). Its protein product is dephosphorylated by calcineurin, which is associated with LVH in human study [33]. *RAI14* (retinoic acid induced protein 14) gene may contribute to the inhibition of adipogenesis by retinoic acid [34]. *MIER3* (mesoderm induction early response 3) gene has been suggested to be candidate breast cancer susceptibility gene [35]. *RP1-272 J12.1* is an uncharacterized gene. Maria et al. presented that *CD36* may impact cardiovascular disease [36]. However, all had no significant association with LVH in our study.

The present study has several limitations. First, as we evaluated several candidate SNPs from the HyperGEN study performed in the western populations, common functional variants in the Korean population may not have been tagged. Differences in linkage disequilibrium between Asian and Western subjects suggest that tracking of causal variant may not be possible. Although this study showed a borderline association between some candidate SNPs and LVH, other causal variants may exist in Asian subjects. If it is considered that the effect of single SNP is small, there might be a possibility that the present study is underpowered. Second, the current results cannot be generalized because they come from adults aged >40 years in a rural area of Korea. Also, echocardiographic data were measured by a single sonographer. Therefore, the intra-class correlation coefficient was not available in the present study. Additionally, we did not investigate the class and period of antihypertensive drugs. Angiotensin-converting enzyme inhibitors and angiotensin-receptor blockers may influence the degree of LVH.

## Conclusions

We analyzed nine candidate SNPs for LVH in the Korean population. The genetic variation in *LOC100507065* (rs4129218 on chromosome 12) had borderline association with LVH, —consistent with findings in Caucasian populations. Although the function of the *LOC100507065* gene product is unknown, it may play a role in LVH development, independent of ethnic background. Further investigation is needed to determine the underlying mechanisms and causal relationships thereof.

## Additional file

Additional file 1: Table S1A. Association analyses between left ventricular mass indexed by BSA and nine candidate SNPs from the HyperGEN study in non-hypertensive population (n=911). **Table S1B.** Association analyses between left ventricular mass indexed by BSA and nine candidate SNPs from the HyperGEN study in hypertensive population (n=726). **Table S2A.** Association analyses between left ventricular mass indexed by height^2.7^ and nine candidate SNPs from the HyperGEN study in non-hypertensive population (n=911). **Table S2B.** Association analyses between left ventricular mass indexed by height^2.7^ and nine candidate SNPs from the HyperGEN study in hypertensive population (n=726).

## Abbreviations

ASE: American Society of Echocardiography
BMI: Body mass index
BP: Blood pressure
BSA: Body surface area
KoGES: Korean Genome Epidemiology Study
LVH: Left ventricular hypertrophy
LVMI: Left ventricular mass index
LVWT: Left ventricular wall thickness
MAF: Minor allele frequency
MRCohort: Multi-Rural Communities Cohort Study
SNP: Single nucleotide polymorphisms
VDR: Vitamin D receptor
WC: Waist circumference
