# Supplementary material for: Validation study of candidate single nucleotide polymorphisms associated with left ventricular hypertrophy in the Korean population
Source: BMC Med Genet. 2015 Mar 15;16:13. doi: 10.1186/s12881-015-0158-1 (PMC4422470; doi:10.1186/s12881-015-0158-1)
Supplement: Additional file 1: Table S1A. — Association analyses between left ventricular mass indexed by BSA and nine candidate SNPs from the HyperGEN study in non-hypertensive population (n=911). Table S1B. Association analyses between left ventricular mass indexed by BSA and nine candidate SNPs from the HyperGEN study in hypertensive population (n=726). Table S2A. Association analyses between left ventricular mass indexed by height2.7 and nine candidate SNPs from the HyperGEN study in non-hypertensive population (n=911). Table S2B. Association analyses between left ventricular mass indexed by height2.7 and nine candidate SNPs from the HyperGEN study in hypertensive population (n=726). [file 12881_2015_158_MOESM1_ESM.doc]

**Additional file 1: Table S1A. Association analyses between left ventricular mass indexed by BSA and nine candidate SNPs from the HyperGEN study in non-hypertensive population (n=911)**.

|  |  | Log (LVM/BSA) | | |  | LVH | | |
| --- | --- | --- | --- | --- | --- | --- | --- | --- |
| SNP | LVM/BSA, g/m^2^ | Beta§ | 95% CI | *P* value |  | OR§ | 95% CI | *P* value |
| rs409045 |  | -0.007 | -0.039-0.025 | 0.671 |  | 0.773 | 0.515-1.161 | 0.215 |
| TT (n=698) | 91.6 ± 20.7 |  |  |  |  |  |  |  |
| CT + CC (n=213) | 91.7.± 22.2 |  |  |  |  |  |  |  |
| rs6450415 |  | 0.031 | 0.002-0.060 | 0.036 |  | 1.732 | 1.216-2.467 | 0.002 |
| TT (n=619) | 91.0 ± 20.9 |  |  |  |  |  |  |  |
| CT +CC (n=292) | 93.1 ± 21.4 |  |  |  |  |  |  |  |
| rs6961069 |  | -0.007 | -0.035-0.020 | 0.605 |  | 1.236 | 0.881-1.732 | 0.220 |
| TT (n=443) | 91.9 ± 20.9 |  |  |  |  |  |  |  |
| CT + CC (n=468) | 91.4 ± 21.2 |  |  |  |  |  |  |  |
| rs10499859 |  | -0.011 | -0.038-0.017 | 0.439 |  | 1.197 | 0.854-1.678 | 0.296 |
| AA (n=442) | 92.1 ± 20.9 |  |  |  |  |  |  |  |
| AG + GG (n=469) | 91.2 ± 21.2 |  |  |  |  |  |  |  |
| rs4129218 |  | -0.014 | -0.042-0.013 | 0.315 |  | 0.880 | 0.625-1.238 | 0.462 |
| AA (n=523) | 92.3 ± 21.6 |  |  |  |  |  |  |  |
| AG +GG (n=388) | 90.7 ± 20.3 |  |  |  |  |  |  |  |
| rs1155635 |  | 0.018 | -0.011-0.046 | 0.229 |  | 1.027 | 0.722-1.461 | 0.883 |
| AA (n=316) | 91.0 ± 21.4 |  |  |  |  |  |  |  |
| GA + GG (n=595) | 92.0 ± 20.9 |  |  |  |  |  |  |  |
| rs2415872 |  | -0.009 | -0.037-0.018 | 0.515 |  | 0.839 | 0.599-1.175 | 0.307 |
| GG (n=405) | 92.1 ± 21.1 |  |  |  |  |  |  |  |
| GC + CC (n=506) | 91.3 ± 21.1 |  |  |  |  |  |  |  |
| rs756529 |  | 0.008 | -0.020-0.036 | 0.594 |  | 1.176 | 0.828-1.670 | 0.364 |
| AA (n=351) | 91.4 ± 21.2 |  |  |  |  |  |  |  |
| GA + GG (n=560) | 91.8 ± 21.0 |  |  |  |  |  |  |  |
| rs10483186 |  | -0.004 | -0.032-0.024 | 0.769 |  | 0.897 | 0.636-1.265 | 0.534 |
| GG (n=338) | 92.2 ± 21.4 |  |  |  |  |  |  |  |
| GT + TT (n=573) | 91.3 ± 20.9 |  |  |  |  |  |  |  |

§Analysis adjusted for age, gender, body mass index, serum creatinine, systolic blood pressure, heart rate and antihypertensive medication.

Abbreviations: 95% CI, confidence interval lower and upper 95%; LVH, left ventricular hypertrophy defined as LVM/BSA ≥ 116 g/m^2^ in males and ≥ 96 g/m^2^ in females; LVM/BSA, left ventricular mass indexed by body surface area; SNP, single nucleotide polymorphism.

**Additional file 1: Table S1B. Association analyses between left ventricular mass indexed by BSA and nine candidate SNPs from the HyperGEN study in hypertensive population (n=726)**.

|  |  | Log (LVM/BSA) | | |  | LVH | | |
| --- | --- | --- | --- | --- | --- | --- | --- | --- |
| SNP | LVM/BSA, g/m^2^ | Beta§ | 95% CI | *P* value |  | OR§ | 95% CI | *P* value |
| rs409045 |  | -0.043 | -0.080--0.006 | 0.022 |  | 0.784 | 0.544-1.130 | 0.192 |
| TT (n=541) | 101.5 ± 23.5 |  |  |  |  |  |  |  |
| CT + CC (n=185) | 97.4.± 22.0 |  |  |  |  |  |  |  |
| rs6450415 |  | 0.001 | -0.034-0.035 | 0.970 |  | 1.264 | 0.902-1.772 | 0.174 |
| TT (n=499) | 100.3 ± 23.4 |  |  |  |  |  |  |  |
| CT +CC (n=227) | 100.8 ± 22.8 |  |  |  |  |  |  |  |
| rs6961069 |  | 0.010 | -0.023-0.042 | 0.559 |  | 1.147 | 0.835-1.575 | 0.397 |
| TT (n=365) | 100.0 ± 22.1 |  |  |  |  |  |  |  |
| CT + CC (n=361) | 100.9 ± 24.3 |  |  |  |  |  |  |  |
| rs10499859 |  | 0.013 | -0.019-0.046 | 0.416 |  | 1.160 | 0.844-1.593 | 0.361 |
| AA (n=357) | 99.9 ± 22.2 |  |  |  |  |  |  |  |
| AG + GG (n=369) | 101.0 ± 24.2 |  |  |  |  |  |  |  |
| rs4129218 |  | -0.030 | -0.063-0.003 | 0.070 |  | 0.701 | 0.504-0.975 | 0.035 |
| AA (n=452) | 101.5 ± 23.7 |  |  |  |  |  |  |  |
| AG +GG (n=274) | 98.7 ± 22.3 |  |  |  |  |  |  |  |
| rs1155635 |  | -0.031 | -0.066-0.004 | 0.081 |  | 0.807 | 0.573-1.135 | 0.218 |
| AA (n=230) | 102.2 ± 22.4 |  |  |  |  |  |  |  |
| GA + GG (n=493) | 99.6 ± 23.6 |  |  |  |  |  |  |  |
| rs2415872 |  | -0.017 | -0.049-0.015 | 0.302 |  | 0.858 | 0.626-1.177 | 0.342 |
| GG (n=330) | 101.2 ± 23.0 |  |  |  |  |  |  |  |
| GC + CC (n=396) | 99.9 ± 23.4 |  |  |  |  |  |  |  |
| rs756529 |  | 0.021 | -0.012-0.055 | 0.213 |  | 1.246 | 0.894-1.737 | 0.195 |
| AA (n=258) | 98.3 ± 21.9 |  |  |  |  |  |  |  |
| GA + GG (n=468) | 101.6 ± 23.9 |  |  |  |  |  |  |  |
| rs10483186 |  | -0.009 | -0.042-0.024 | 0.590 |  | 1.102 | 0.797-1.522 | 0.558 |
| GG (n=284) | 101.0 ± 24.1 |  |  |  |  |  |  |  |
| GT + TT (n=442) | 100.1 ± 22.7 |  |  |  |  |  |  |  |

§Analysis adjusted for age, gender, body mass index, serum creatinine, systolic blood pressure, heart rate and antihypertensive medication.

Abbreviations: 95% CI, confidence interval lower and upper 95%; LVH, left ventricular hypertrophy defined as LVM/BSA ≥ 116 g/m^2^ in males and ≥ 96 g/m^2^ in females; LVM/BSA, left ventricular mass indexed by body surface area; SNP, single nucleotide polymorphism.

**Additional file 1: Table S2A. Association analyses between left ventricular mass indexed by height^2.7^ and nine candidate SNPs from the HyperGEN study in non-hypertensive population (n=911).**

|  |  | Log (LVM/height^2.7^) | | |  | LVH | | |
| --- | --- | --- | --- | --- | --- | --- | --- | --- |
| SNP | LVM/height^2.7^, g/m^2.7^ | Beta§ | 95% CI | *P* value |  | OR§ | 95% CI | *P* value |
| rs409045 |  | -0.005 | -0.038-0.028 | 0.759 |  | 0.784 | 0.539-1.139 | 0.202 |
| TT (n=698) | 42.7 ± 10.5 |  |  |  |  |  |  |  |
| CT + CC (n=213) | 43.2 ± 11.6 |  |  |  |  |  |  |  |
| rs6450415 |  | 0.033 | 0.004-0.063 | 0.028 |  | 1.516 | 1.093-2.105 | 0.013 |
| TT (n=619) | 42.6 ± 10.7 |  |  |  |  |  |  |  |
| CT +CC (n=292) | 43.5 ± 10.9 |  |  |  |  |  |  |  |
| rs6961069 |  | -0.007 | -0.035-0.021 | 0.634 |  | 1.114 | 0.817-1.519 | 0.496 |
| TT (n=443) | 43.1 ± 10.3 |  |  |  |  |  |  |  |
| CT + CC (n=468) | 42.7 ± 11.1 |  |  |  |  |  |  |  |
| rs10499859 |  | -0.011 | -0.039-0.017 | 0.450 |  | 1.101 | 0.807-1.501 | 0.543 |
| AA (n=442) | 43.2 ± 10.3 |  |  |  |  |  |  |  |
| AG + GG (n=469) | 42.6 ± 11.1 |  |  |  |  |  |  |  |
| rs4129218 |  | -0.018 | -0.046-0.010 | 0.198 |  | 0.798 | 0.582-1.093 | 0.160 |
| AA (n=523) | 43.4 ± 11.2 |  |  |  |  |  |  |  |
| AG +GG (n=388) | 42.1 ± 10.1 |  |  |  |  |  |  |  |
| rs1155635 |  | 0.015 | -0.014-0.044 | 0.319 |  | 1.004 | 0.724-1.391 | 0.982 |
| AA (n=316) | 42.6 ± 10.5 |  |  |  |  |  |  |  |
| GA + GG (n=595) | 43.0 ± 10.8 |  |  |  |  |  |  |  |
| rs2415872 |  | -0.010 | -0.038-0.018 | 0.479 |  | 0.956 | 0.701-1.305 | 0.779 |
| GG (n=405) | 43.0 ± 10.6 |  |  |  |  |  |  |  |
| GC + CC (n=506) | 42.7 ± 10.9 |  |  |  |  |  |  |  |
| rs756529 |  | 0.006 | -0.023-0.034 | 0.705 |  | 1.037 | 0.754-1.427 | 0.821 |
| AA (n=351) | 42.7 ± 10.9 |  |  |  |  |  |  |  |
| GA + GG (n=560) | 43.0 ± 10.7 |  |  |  |  |  |  |  |
| rs10483186 |  | -0.002 | -0.031-0.027 | 0.901 |  | 0.931 | 0.678-1.278 | 0.658 |
| GG (n=338) | 43.2 ± 10.6 |  |  |  |  |  |  |  |
| GT + TT (n=573) | 42.7 ± 10.8 |  |  |  |  |  |  |  |

§Analysis adjusted for age, gender, body mass index, serum creatinine, systolic blood pressure, heart rate and antihypertensive medication.

Abbreviations: 95% CI, confidence interval lower and upper 95%; LVH, left ventricular hypertrophy defined as LVM/ height^2.7^ ≥ 49 g/m^2.7^ in males and ≥ 45 g/m^2.7^ in females; LVM/height^2.7^, left ventricular mass indexed by height^2.7^; SNP, single nucleotide polymorphism.

**Additional file 1: Table S2B. Association analyses between left ventricular mass indexed by height^2.7^ and nine candidate SNPs from the HyperGEN study in hypertensive population (n=726).**

|  |  | Log (LVM/height^2.7^) | | |  | LVH | | |
| --- | --- | --- | --- | --- | --- | --- | --- | --- |
| SNP | LVM/height^2.7^, g/m^2.7^ | Beta§ | 95% CI | *P* value |  | OR§ | 95% CI | *P* value |
| rs409045 |  | -0.046 | -0.084--0.008 | 0.019 |  | 0.808 | 0.558-1.171 | 0.260 |
| TT (n=541) | 49.1 ± 12.8 |  |  |  |  |  |  |  |
| CT + CC (n=185) | 47.3 ± 11.6 |  |  |  |  |  |  |  |
| rs6450415 |  | -0.008 | -0.044-0.027 | 0.649 |  | 1.107 | 0.782-1.567 | 0.567 |
| TT (n=499) | 48.6 ± 12.5 |  |  |  |  |  |  |  |
| CT +CC (n=227) | 48.7 ± 12.5 |  |  |  |  |  |  |  |
| rs6961069 |  | 0.012 | -0.021-0.045 | 0.477 |  | 1.154 | 0.834-1.595 | 0.388 |
| TT (n=365) | 48.1 ± 11.9 |  |  |  |  |  |  |  |
| CT + CC (n=361) | 49.2 ± 13.1 |  |  |  |  |  |  |  |
| rs10499859 |  | 0.017 | -0.016-0.050 | 0.323 |  | 1.205 | 0.871-1.666 | 0.261 |
| AA (n=357) | 48.1 ± 11.9 |  |  |  |  |  |  |  |
| AG + GG (n=369) | 49.2 ± 13.1 |  |  |  |  |  |  |  |
| rs4129218 |  | -0.034 | -0.068-0.000 | 0.051 |  | 0.672 | 0.481-0.941 | 0.021 |
| AA (n=452) | 49.1 ± 12.6 |  |  |  |  |  |  |  |
| AG +GG (n=274) | 47.9 ± 12.4 |  |  |  |  |  |  |  |
| rs1155635 |  | -0.032 | -0.068-0.004 | 0.080 |  | 0.732 | 0.514-1.041 | 0.082 |
| AA (n=230) | 49.1 ± 12.0 |  |  |  |  |  |  |  |
| GA + GG (n=493) | 48.4 ± 12.8 |  |  |  |  |  |  |  |
| rs2415872 |  | -0.014 | -0.047-0.019 | 0.419 |  | 0.912 | 0.660-1.261 | 0.578 |
| GG (n=330) | 49.2 ± 12.7 |  |  |  |  |  |  |  |
| GC + CC (n=396) | 48.2 ± 12.3 |  |  |  |  |  |  |  |
| rs756529 |  | 0.022 | -0.013-0.057 | 0.213 |  | 1.061 | 0.757-1.488 | 0.729 |
| AA (n=258) | 47.7 ± 11.8 |  |  |  |  |  |  |  |
| GA + GG (n=468) | 49.2 ± 12.9 |  |  |  |  |  |  |  |
| rs10483186 |  | -0.010 | -0.044-0.023 | 0.545 |  | 0.953 | 0.686-1.325 | 0.953 |
| GG (n=284) | 48.7 ± 12.9 |  |  |  |  |  |  |  |
| GT + TT (n=442) | 48.6 ± 12.3 |  |  |  |  |  |  |  |

§Analysis adjusted for age, gender, body mass index, serum creatinine, systolic blood pressure, heart rate and antihypertensive medication.

Abbreviations: 95% CI, confidence interval lower and upper 95%; LVH, left ventricular hypertrophy defined as LVM/ height^2.7^ ≥ 49 g/m^2.7^ in males and ≥ 45 g/m^2.7^ in females; LVM/height^2.7^, left ventricular mass indexed by height^2.7^; SNP, single nucleotide polymorphism.
